# Explaining gender differences in non-fatal suicidal behaviour among adolescents: a population-based study

**DOI:** 10.1186/1471-2458-11-597

**Published:** 2011-07-28

**Authors:** Michael Kaess, Peter Parzer, Johann Haffner, Rainer Steen, Jeanette Roos, Martin Klett, Romuald Brunner, Franz Resch

**Affiliations:** 1 Department of Child and Adolescent Psychiatry, Centre of Psychosocial Medicine, University of Heidelberg, Heidelberg, Germany; 2 Public Health Department of Heidelberg, Heidelberg, Germany; 3 Department of Educational Psychology, University of Education Heidelberg, Heidelberg, Germany; 4 Orygen Youth Health Research Centre, The University of Melbourne, Melbourne, Australia

**Keywords:** suicidal behaviour, suicide, gender, adolescence, emotional and behaviour problems

## Abstract

**Background:**

While suicide is the second leading cause of death among young people in most industrial countries, non-fatal suicidal behaviour is also a very important public health concern among adolescents. The aim of this study was to investigate gender differences in prevalence and emotional and behavioural correlates of suicidal behaviour in a representative school-based sample of adolescents.

**Methods:**

A cross-sectional design was used to assess suicidal behaviour and various areas of emotional and behavioural problems by using a self-report booklet including the Youth Self-Report. One hundred sixteen schools in a region of Southern Germany agreed to participate. A representative sample of 5,512 ninth-grade students was studied. Mean age was 14.8 years (SD 0.73); 49.8% were female.

**Results:**

Serious suicidal thoughts were reported by 19.8% of the female students and 10.8% of the females had ever attempted suicide. In the male group, 9.3% had a history of suicidal thoughts and 4.9% had previously attempted suicide. Internalizing emotional and behavioural problems were shown to be higher in the female group (difference of the group means 4.41) while externalizing emotional and behavioural problems slightly predominated in male students (difference of the group means -0.65). However, the total rate of emotional and behavioural problems was significantly higher in the adolescent female group (difference of the group means 4.98). Using logistic regression models with suicidal thoughts or attempted suicide as dependent variables, the pseudo-R^2^ of gender alone was only 2.7% or 2.3%, while it was 30% or 23.2% for emotional and behavioural problems measured by the YSR syndrome scales. By adding gender to the emotional and behavioural problems only an additional 0.3% of information could be explained.

**Conclusions:**

The findings suggest that gender differences in non-fatal suicidal behaviour among adolescents can to a large extent be explained by the gender differences in emotional and behavioural problems during this age.

## Background

Suicide and non-fatal suicidal behaviour are both well-recognized public health problems in young people [1,2]. Whereas the prevalence of suicide and suicidal behaviour remains relatively low before puberty [3], adolescent suicide is one of the leading causes of death in the teenaged group [4]. In Europe, suicide is the second leading cause of death in male and female adolescents [5]. In the USA, suicide is reported to be the third leading cause of death after accidents and homicides [6]. According to the findings of a WHO multi-centre study conducted in young people 15 to 24 years of age, the increase in suicide has been shown to be associated with an increase in suicide attempts [7]. Results of a systematic review of 128 studies on the prevalence of suicidal phenomena in adolescents revealed that, on average, 9.7% (95% CI 8.5 to 10.9) of adolescents reported to have attempted suicide while even 29.9% (95% CI 26.1 to 33.8) of these adolescents indicated having thought about suicide at some point in their life [8].

One of the strongest predictors of completed suicide or further suicide attempt has been found to be a previous suicide attempt [9,10]. Therefore, suicide attempts and also suicidal thoughts in adolescents must always be taken seriously, in spite of the fact that for every death of a young person from suicide, many suicide attempts have already been undertaken, especially by girls [9,11]. In most western countries, females are more likely to engage in suicidal behaviour, but are less likely to die as a result of a suicidal act than males [12-14]. This "gender paradox" is known to be extremely distinctive in adolescents; during this period of life suicide attempts are 3-9 times more common in girls while completed suicides rates are 2-4 times higher in adolescent males [15]. Many epidemiological studies so far have reported higher rates of non-fatal suicidal behaviour in females which could indicate a gender-specific predisposition for the experience of suicidal thoughts and suicide attempts during this life-period [6,15,16].

As regards mental illness as one of the strongest risk factors for suicidal behaviour, depression and anxiety in particular seem to function as mediators of adolescent suicidal behaviour [17]. But drug abuse, risk behaviours [6] and other types of externalizing psychopathological behaviours [18] are also associated with an increased risk of suicide among adolescents.

There is only little empirical research which investigated the reasons underlying the correlation of being female and showing higher rates of suicidal thoughts and suicide attempts during adolescence. One theory is that gender differences in psychopathology could play an important role in this issue [12,19,20], suggesting that different types and characteristics of emotional and behavioural problems may primarily lead to unequal rates of suicidal behaviour in female and male individuals. Therefore, our hypothesis was that gender differences in non-fatal suicidal behaviour among adolescents could mainly be explained by the gender differences in emotional and behavioural problems.

## Methods

### Study population and design

The Heidelberg School Study, a cross-sectional, nationally representative, school-based sample was devised to measure the prevalence and common psychological correlates of non-fatal suicidal behaviour, self-destructive behaviour and other forms of risk behaviour in adolescents. The investigation took place in cooperation with the Heidelberg Public Health Service and the University of Education between October 2004 and January 2005. All schools in the Rhein-Neckar District were invited to participate. The Rhein-Neckar District is typical for geographically mixed populations in Germany and therefore shows a representative distribution of types of schools and parental socioeconomic status [21]. Of 121 schools contacted (N = 6,842), 116 agreed to participate. Five schools declined participation without giving reasons. All ninth-grade students of the 116 participating schools (N = 6,534) were requested to take part in the study; 349 students were absent on the day of the assessment, 100 students did not return their questionnaire (N = 6,085). Additionally, 573 students had missing data (N = 5,512). Hence, we could include data for 89.1% of the students who were approached and for 80.6% of the entire population of ninth-grade students in this school district [22,23].

### Study procedures

The Heidelberg School Study was performed in compliance with the Helsinki Declaration. It was appraised and approved by the ethics committee of the faculty of medicine at the University of Heidelberg. While parents or legal guardians were informed about the study by letter about 4 weeks before it commenced, informed and written consent was obtained from all participating students. The student's anonymity and voluntary status were ensured before starting the investigation. Data collectors, who had been trained in test administration by the research team, administered the questionnaire to all participating subjects during a regular class period. Although teachers were in attendance during this class period for legal reasons, they did not interact with the students at any time during the assessment. For adolescents in special education classes, the survey personnel assisted subjects who had problems filling out the questionnaire and offered them additional time to complete it. However, this kind of support was only necessary in a few cases.

### Assessment

Students were given a self-report booklet, including diverse questions regarding socio-demographic characteristics as well as items comprising suicidal and self-destructive behaviour, different forms of risk behaviour, and emotional and behavioural problems. Suicidal behaviour was assessed by pertinent parts of the German version [24] of the Schedule for Affective Disorders and Schizophrenia for School-Age Children [25] that had been inserted into the self-report booklet. Suicidal ideation was assessed by the question "Did you ever seriously think about taking your own life?" Suicide attempts were assessed by the question "Have you ever tried to take your own life?" A separate question on self-harm (e.g. cutting, burning, etc.) was also asked during the survey and has been published elsewhere [22], therefore the students were probably able to distinguish between self-harm with or without the intent to die. All variables were assessed as binominal variables with the categories "yes" or "no". To assess a broad range of emotional and behavioural problems potentially associated with suicidal behaviour, the German version [26] of the Youth Self-Report (YSR) [27], a self-report version of the Child Behaviour Checklist (CBCL) [28], was administered. This self-report questionnaire consists of 8 scales, including withdrawn (YSR 1), somatic complaints (YSR 2), anxious/depressed (YSR 3), social problems (YSR 4), thought problems (YSR 5), attention problems (YSR 6), delinquent behaviour (YSR 7), aggressive behaviour (YSR 8), and the summary scales of internalizing and externalizing problems as well as an YSR total score of emotional and behavioural problems.

### Statistical analyses

Gender differences in the YSR syndrome scales were tested with t-tests. To analyse the association of gender and emotional and behavioural problems with suicidal thoughts and suicide attempts, a sequence of logistic regression models were estimated for each of the two dependent variables. Emotional and behavioural problems were operationalized by the raw scores of the eight syndrome scales of the YSR. For the assessment of the goodness of fit of the models two different measures were used. The pseudo-R^2^is the fraction of information of the dependent variable that is explained by the independent variables [29]. The Bayes Information Criterion (BIC) allows comparison of models according to their estimated ability to predict new data [30]. The model with the minimum BIC is the best model to predict future data. To test the hypotheses that one model is significant better than another we used the likelihood-ratio test (LR-test). For all calculations the statistical software Stata 11 was used.

## Results

Table 1 presents the sample classified into separate male and female groups as well as the total amount of participating students. For each of these groups, the table reports socio-demographic variables, school types and familial background. Suicidal thoughts were reported by 14.51% of the students, while there were higher rates in female (19.80%) than in male (9.28%) adolescents. A history of at least one suicide attempt was found in 7.84% of the participants, but again, in 10.83% of the girls and only in 4.88% of the boys. In summary, the prevalence rates of suicidal behaviour in female students were twice as high as the prevalence rates for the male students suggesting a strong influence of gender on suicidal thoughts and suicide attempts.

**Table 1:** Sociodemographic variables, school type, familial background, and suicidal behaviour.

Characteristics	Females (n = 2,743)	Males (n = 2,769)	Total (n = 5,512)
Age, mean (SD), y	14.77 (0.72)	14.88 (0.74)	14.82 (0.73)
Nationality, %			
German	89.49	87.90	88.69
Other	10.51	12.10	11.31
School type*, %			
Gymnasium	38.79	32.83	35.79
Realschule	33.25	31.92	32.58
Hauptschule	25.41	32.18	28.81
Förderschule	2.55	3.07	2.81
Family composition, %			
2 Parents	74.08	76.14	75.11
1 Parent	14.76	14.47	14.61
1 Parent and a partner	9.90	8.19	9.05
Other	1.27	1.20	1.23

* After four years of elementary school the German school system branches into three types of secondary schools. The so called "Hauptschule" (Secondary General School which takes five years after Primary School) prepares pupils for vocational training, whereas the "Realschule" (Intermediate Secondary School) concludes with a general certificate of secondary education after six years. Eight years of "Gymnasium" provide pupils with a general university entrance qualification

Mean scores of behavioural and emotional problems as measured by the YSR scales are presented in table 2. Female students reported significantly higher scores on the scales measuring withdrawn, somatic complaints, anxious/depressed, thought problems, attention problems, as well as in the score for internalizing problems, and also in the YSR total score (p < .01 for all). For male students we found elevated scores in social problems, delinquent behaviour, and in the score for externalizing problems (p < .01 for all). The only scale that showed no significant gender difference was aggressive behaviour.

**Table 2:** Mean (SD) for the YSR scores and the gender differences with 95%-confidence intervals.

YSR scales	Female	Male	Total	Gender difference	Confidence interval
1. Withdrawn	3.36 (2.45)	2.66 (2.26)	3.01 (2.38)	0.69	0.57 - 0.82
2. Somatic complaints	3.75 (2.90)	2.11 (2.15)	2.93 (2.67)	1.64	1.50 - 1.77
3. Anxious/depressed	6.78 (4.89)	4.40 (3.80)	5.59 (4.53)	2.38	2.15 - 2.61
4. Social problems	1.97 (1.95)	2.13 (1.93)	2.05 (1.94)	-0.16	-0.27 - -0.06
5. Thought problems	1.59 (1.97)	1.31 (1.89)	1.45 (1.94)	0.27	0.17 - 0.38
6. Attention problems	4.81 (2.70)	4.34 (2.74)	4.57 (2.73)	0.47	0.32 - 0.61
7. Delinquent behaviour	4.21 (3.00)	4.79 (3.28)	4.50 (3.16)	-0.59	-0.75 - -0.42
8. Aggressive behaviour	8.74 (4.76)	8.81 (5.64)	8.77 (5.22)	-0.07	-0.34 - 0.21

Internalizing problems	13.34 (8.11)	8.92 (6.47)	11.12 (7.66)	4.41	4.02 - 4.80
Externalizing problems	12.95 (6.99)	13.60 (8.19)	13.27 (7.62)	-0.65	-1.06 - -0.25

YSR Total Score	40.33 (19.26)	35.35 (18.55)	37.82 (19.07)	4.98	3.98 - 5.98

Goodness of fit for the sequence of logistic regression models to predict suicidal thoughts is summarized in table 3. Gender alone explains 2.7% of the information (model 1), while emotional and behavioural problems explain 30% (model 2). By adding gender to the emotional and behavioural problems only an additional 0.3% of information can be explained (model 3). This small amount is still significant due to the large sample size (LR-test of model 2 vs. model 3: χ^2^(1) = 12.31, p = .0004). This model is also the one with the smallest BIC and therefore is the one that we should use to predict suicidal thoughts. Including the interaction of gender with emotional and behavioural problems in model 4 - corresponding to the hypothesis that the association of emotional and behavioural problems with suicidal thoughts is different for males and females - does not result in a significant increase in prediction (LR-test of model 3 vs. model 4: χ^2^(8) = 10.2, p = .251). Looking at the estimated coefficients for the best model (model 3), it can be seen that the scales anxious/depressed and delinquent behaviour have the largest odds ratios per unit of raw score (table 4).

**Table 3:** Goodness of fit of the logistic regression models for suicidal thoughts and suicide attempts.

Model	suicidal thoughts		suicide attempts	
	Pseudo-R^2^	BIC	Pseudo-R^2^	BIC
1. gender only	.027	4458	.023	2977
2. YSR scales only	.300	3274	.232	2406
3. gender and YSR scales	.303	3270	.235	2404
4. gender, YSR scales and their interaction	.305	3329	.236	2470

**Table 4:** Regression coefficients in their exponential form as odds ratios (OR) and their 95%-confidence intervals (CI).

Coefficient	suicidal thoughts		suicide attempts	
	OR	CI	OR	CI
Female gender	1.454	1.179 - 1.794	1.532	1.179 - 1.990
YSR 1: Withdrawn	1.005	0.956 - 1.057	0.961	0.905 - 1.021
YSR 2: Somatic complaints	1.049	1.012 - 1.088	1.066	1.023 - 1.111
YSR 3: Anxious/depressed	1.298	1.259 - 1.338	1.195	1.156 - 1.235
YSR 4: Social problems	0.880	0.833 - 0.929	0.885	0.828 - 0.945
YSR 5: Thought problems	1.086	1.038 - 1.137	1.062	1.010 - 1.117
YSR 6: Attention problems	0.975	0.933 - 1.019	1.018	0.966 - 1.073
YSR 7: Delinquent behaviour	1.210	1.168 - 1.253	1.171	1.125 - 1.218
YSR 8: Aggressive behaviour	0.982	0.960 - 1.005	1.009	0.982 - 1.036

OR are presented for the models with the best fit for suicidal thoughts and suicide attempts (model 3 for both). For the YSR scales the OR is the ratio of the odds for one point of increase in the raw score.

The results for suicide attempts are very similar (table 3). Here again the percentage of information explained by gender alone (model 1: 2.3%) is much smaller than the percentage explained by the YSR scales alone (model 2: 23.2%). The information explained by gender in addition to the emotional and behavioural problems is with 0.3% (model 3) again without practical relevance but statistically significant (LR-test of model 2 vs. model 3: χ^2^(1) = 10.37, p = .0013). The lack of interaction between gender and emotional and behavioural problems (LR-test of model 3 vs. model 4: χ^2^(8) = 2.65, p = .955) indicates, that the influence of emotional and behavioural problems on the probability of suicide attempts is equal for girls and boys. Also the pattern of the regression coefficients for suicide attempts is comparable to the one for suicidal thoughts (table 4).

## Discussion

In the present study, the prevalence rates of suicidal behaviour, divided into the two categories "suicidal thoughts" and "suicide attempts", were high among adolescent students in Germany but in line with previous epidemiological research [8]. Within the sample as a whole a considerably higher prevalence of both suicidal thoughts and suicide attempts could be found in girls than in boys. These results are in line with many other authors who reported about two to four times higher rates of suicidal thoughts and suicide attempts in female than in male adolescents [6,12,31,32]. Similar findings may also occur in nonsuicidal self-harming behaviour among adolescents as Brunner and colleagues have previously reported quite similar gender distributions and an important role of mediating depression for repetitive self-harm in this sample [22]. Such similarities might be of interest for the ongoing discussion if self-harming and suicidal behaviour are both on the same continuum of self-destructive behaviour.

Concerning emotional and behavioural problems as indicators for an existing psychopathology, female students showed higher levels of internalizing problems whereas male students tended to have slightly higher degrees of externalizing problems as assessed with the YSR. These results are not surprising as it is well known that females usually show higher rates of internalizing disorders while males tend to suffer from externalizing emotional and behavioural problems [18-20,33]. Given these differences in internalizing and externalizing psychopathology, it could be hypothesized that boys tend to act out their personal problems and therefore are more likely to show aggressive (externalizing) behaviours while girls more often show auto-aggressive (internalizing) symptomatology.

An important finding of our study is that the gender effect is more distinct for internalizing emotional and behavioural problems with far higher rates of internalizing problems in female students and males presenting only a marginally higher rate of externalizing problems. Female adolescents did not only show considerably higher values for somatic complaints, depression and anxiety but also suffered from more emotional and behavioural problems on the whole. A possible explanation for the fact that female adolescents suffer from more emotional and behavioural problems than same-aged males could be the significant margin of adolescent development in females during this period. It is well known that psychopathology is rare during childhood and increases during adolescence [34]; thus, females aged between 14 and 16 are usually further along in their development and therefore might present with more emotional and behavioural problems than do males. Suicidal behaviour might then increase later in male adolescents' lives which already could be demonstrated in previous research [35]. As delinquency is also highly associated with suicidal behaviour, it could be hypothesized that this consequently might be the more male pathway into suicidal behaviour. Additionally, some authors have argued that females are more likely to communicate their problems to others and to engage in help-seeking behaviours but at the same time they are also more likely to ruminate about problems they encounter which can affect their wellbeing, and consequently might be a cause for gender differences especially in internalizing problems [2]. Another possible explanation for differences in both emotional problems and suicidal behaviour might be different experiences of childhood adversities, as e.g. childhood sexual abuse is much more common in girls and is also strongly related to depression, self-harm and suicidal behaviour during adolescence and adulthood [36]. A more neurobiological approach to explain gender differences in psychopathology has previously been reported by Schneider and colleagues who found differences in emotion processing that might account for such gender differences [37].

Our findings suggest that there is an exceptionally strong association for depression, anxiety and delinquent behaviour with suicidal behaviour. This is consistent with findings from many other studies that reported emotional and behavioural problems to be strongly associated with suicidal behaviour and gave evidence for a correlation of suicidal thoughts and suicide attempts with both internalizing and externalizing problems [20,38-42].

The main purpose of this study was to investigate potential gender differences in emotional and behavioural problems and the extent to which these are predictive of adolescent non-fatal suicidal behaviour. Our results clearly demonstrate that gender alone does only explain 2.3 -2.7% of the information of non-fatal suicidal behaviour, while emotional and behavioural problems explain 23.2 -30% of the information. By adding gender to the emotional and behavioural problems only an additional 0.3% of information can be explained, which, however, is still significant due to the large sample size, but practically hardly relevant. This finding may strongly support the hypothesis that explanations for gender differences in rates of suicidal behaviour may arise from gender related differences in psychopathology [12], as it is in line with findings of Fergusson and colleagues who also supported this theory [19]. However, it has to be acknowledged that, regarding the whole model of suicidal behaviour, there are many additional influencing factors which could not be included in this analysis. Nevertheless, adolescent psychopathology seems to be responsible for the by far largest portion of gender differences.

Overall, the results of our study support the view that girls clearly are at higher risk of engaging in non-fatal suicidal behaviour during adolescence. Notably, emotional and behavioural problems during this period in life could play an important role in this regard. In conclusion, girls may be more likely to exhibit suicidal behaviour because they suffer from a higher rate of emotional and behavioural problems during adolescence. This finding could to a large extend explain the existing gender differences in suicidal behaviour and should be considered as one main reason for these gender differences [13,19,43]. However, we still need to understand the missing link between gender and adolescent psychopathology which could range from genetic and neurobiological aspects to adverse childhood experiences and other environmental factors.

The overall interpretation of our findings is limited by the cross-sectional design of the study. Therefore it is not possible to determine whether the elevated rates of emotional and behavioural problems are the cause rather than the consequence of suicidal behaviour. A possible causal relationship can only be investigated in a longitudinally designed study. However, the fact that the investigation took place at the onset of suicidal behaviour -- as there is evidence that suicidal behaviour begins in early to middle adolescence -- could at least alleviate this limitation. One additional problem of this cross-sectional design relying on self-report questionnaires might be that higher reported prevalence rates of suicidal behaviour in girls could also have occurred due to self-report bias influenced by their current mood stages. However, this bias mainly occurs during self-report assessments of adverse childhood experiences or life events which usually try to assess quite subjective appraisal. In contrast, our questions on suicidal thoughts and particularly suicidal acts seem to be more distinct and therefore less prone to self-report bias.

The strength of this cross-sectional design is its anonymity, which heightened the acceptance of all involved parties and was a prerequisite on the part of the school authorities. Several factors strengthen the validity of the findings reported in this study. First, our sample was representative of ninth-grade students in Germany as we had a large sample and a low dropout rate. Secondly, we used a clear definition of suicidal behaviour, divided into suicidal ideation and suicide attempts. Thirdly, adolescents' reports may be of enhanced validity as this survey was based on a self-rating conducted anonymously.

## Conclusions

From the results of our study we could draw the following main conclusions:

First, there are remarkable gender differences in both, adolescent non-fatal suicidal behaviour and emotional and behavioural problems. Females are more likely to be engaged in non-fatal suicidal behaviour and to suffer from emotional and behavioural problems during adolescence.

Second, different occurrences and extents of emotional and behavioural problems in females and males might explain the gender differences in suicidal behaviour to a large extent. These findings may support the differential psychopathology theory, which explains gender differences in terms of occurrence of suicidal thoughts and non-fatal suicide attempts by different rates of psychopathology with females suffering more from internalizing disorders [13,20,43].

Our results indicate that female adolescents are at specific risk of experiencing non-fatal suicidal behaviour. They emphasize the importance of non-fatal suicidal behaviour during adolescence as an issue of public health and clearly distinguish the need to consider female and male adolescents separately concerning their risk for suicidal behaviour and, particularly, the leading psychopathological characteristics of suicidal behaviour. Additionally, the findings clearly show that the higher rate of psychopathology in female adolescents is strongly associated with suicidal behaviour and therefore may possibly represent a main aetiological factor as compared to males. Therefore, either detection or prevention and treatment of suicidal behaviour in adolescents should especially focus on the gender differences in emotional and behavioural problems during adolescence.

## Competing interests

The authors declare that they have no competing interests.

## Authors' contributions

MK designed the analysis plan, analysed the data, interpreted the findings and wrote the first draft of this paper. PP and RB helped develop the analysis plan, analysed the data, and helped interpret the findings. JH had the idea of the study. MK, PP, RS, JR, MK, RB and FR helped design and implement the study. MK and FR obtained funding. All authors contributed to the writing of this paper and read and approved the final manuscript. MK is the guarantor.

## Pre-publication history

The pre-publication history for this paper can be accessed here:

http://www.biomedcentral.com/1471-2458/11/597/prepub
